# Combination of wet fixation and drying treatments to improve dye fixation onto spray-dyed cotton fabric

**DOI:** 10.1038/s41598-021-94885-z

**Published:** 2021-07-28

**Authors:** Lina Lin, Wenju Zhu, Cong Zhang, Md. Yousuf Hossain, Zubair Bin Sayed Oli, Md. Nahid Pervez, Shamima Sarker, Md. Ikram Ul Hoque, Yingjie Cai, Vincenzo Naddeo

**Affiliations:** 1 Hubei Provincial Engineering Laboratory for Clean Production and High Value Utilization of Bio-Based Textile Materials, Wuhan Textile University, Wuhan, 430200 China; 2 Engineering Research Centre for Clean Production of Textile Dyeing and Printing, Ministry of Education, Wuhan Textile University, Wuhan, 430200 China; 3 Sanitary Environmental Engineering Division (SEED), Department of Civil Engineering, University of Salerno, via Giovanni Paolo II 132, 84084 Fisciano, SA Italy; 4 College of Material Science and Engineering, Donghua University, Shanghai, 201620 China; 5 Discipline of Chemistry, The University of Newcastle, University Drive, Callaghan, NSW 2308 Australia; 6 Australian Institute for Bioengineering and Nanotechnology (AIBN), The University of Queensland, Brisbane, QLD 4072 Australia

**Keywords:** Chemical engineering, Materials chemistry

## Abstract

The conventional dyeing process requires a substantial amount of auxiliaries and water, which leaches hazardous colored effluents to the environment. Herein, a newly developed sustainable spray dyeing system has been proposed for cotton fabric in the presence of reactive dyes, which has the potential to minimize the textile dyeing industries environmental impact in terms of water consumption and save significant energy. The results suggest that fresh dye solution can be mixed with an alkali solution before spray dyeing to avoid the reactive dye hydrolysis phenomenon. After that, drying at 60–100 °C, wet fixation treating for 1–6 min, and combined treatments (wet fixation + drying) were sequentially investigated and then dye fixation percentages were around 63–65%, 52–70%, and above 80%, respectively. Following this, fixation conditions were optimized using L_16_ orthogonal designs, including wet fixation time, temperature, dye concentration, and pH with four levels where the “larger-the-better” function was selected to maximize the dye fixation rate. Additionally, the color uniformity and wash and rubbing fastnesses were at an acceptable level when both treatments were applied. Finally, the dyes were hydrolyzed after wet fixation, and the hydrolysis percentages were enhanced after the drying process.

## Introduction

Reactive dye is a popular and effective dyestuff for cotton fabric dyeing due to its useful properties, such as good colorfastness, reproducibility, wide ranges of hues, brilliant color, convenience, and easy application of the dye^1–3^. The traditional reactive dyeing for cotton fabric uses a water bath system because the dye solubility is very high in water^4^. Nevertheless, a massive amount (30%) of reactive dyes remained unused in the dyeing operation and directly discarded into watercourses before any primary treatment. These colored effluents contain complex substances of hydrolyzed reactive dyes, auxiliaries, heavy metals, and organic compounds, which strongly affect the natural ecosystem and human health^5,6^.

There is a growing interest in using innovative, eco-friendly, and sustainable dyeing methods to reduce water use, energy consumption, and hazardous colored effluent discharge. Therefore, there are continuous attempts to develop more efficient dyeing systems that should not only produce less and/or no hazardous effluent but also enhance utilization efficiency of the dye and energy, e.g., liquid ammonia dyeing^7^, supercritical fluid dyeing^8^, crosslinking agents in textile dyeing^9^, low salt/salt-free reactive dyeing^10^, dyeing using non-aqueous dyebaths^11,12^, and nanoparticles-based dyeing^13–15^. While these methods have proven useful for sustainable dyeing system, they have not been shown prospective for large scale applications.

The pad-dyeing process is regarded as a relatively clean dyeing method for reactive dyeing with cotton fabric because of their deep color formation, the higher rate of fixation and affordability^16,17^. Various types of pad-dyeing process are widely used nowadays, such as the pad-batch, pad-steam, pad-dry, and pad-dry-pad-steam. Pad-batch dyeing has many advantages, such as strong versatility, energy savings, economics, and convenience^18^, but it is a semi-continuous and time-consuming process (it need high fixation time) as the dyeing is carried out at room temperature^19^. Pad-steam dyeing is a prudent method, which has the advantage of short processing time since the steaming can be performed continuously, but the process has disadvantages of high water and energy consumption^20^. However, hydrolysis of reactive dyes leads to limited dye build-up behavior, rendering the pad-steam process only suitable for light to medium shades^20^. Similarly, pad-dry-pad-steam is a continuous process, but during the intermediate drying process, it is responsible for high energy consumption and has the possibility of low shade changes and the risk of dye migration during drying^20^. Alternatively, in the exhaust reactive dyeing process, salt is used as an electrolyte to improve the dye uptake, and about 10–50 g L^−1^ salt is used at the dye bath according to different dye concentrations^21^. However, dyeing wastewater contains a large quantity of salts that are of concern since they are difficult to remove.

Spray dyeing technology is applied in jet-type overflow dyeing machine, and the machine is widely used in exhaust dyeing of knit fabric with a low liquid ratio to decrease the water consumption. However, it is used in the batch dyeing process, not continue dyeing process^22^. We designed an innovative continue dyeing system that included preparation of the dye solution, spray dyeing of the fabric, dye fixation treatment, soapy washing of the unfixed dye on the dyed fiber, and drying. This is rendering a promising dyeing system for the textile fabrics by virtue of its simple installation, support to the sustainability solution, no required salt and retained the fabric structure during the operation^23^. Spray dyeing process can eliminate the urea usage in contrast with the conventional pad-batch (50 g L^−1^)^24^ or pad-dry-bake dyeing process (100–200 g L^−1^)^25^, and also can avoid the need for inorganic salt^18^ in the conventional pad-steam process (50 g L^−1^)^26^ or pad-dry-chemical pad-steam process (125–200 g L^−1^)^27^. In addition, a combination of wet fixation and drying treatment for a high dye fixation rate in a short time was applied in this innovative system, which saves dye fixation treating time and reduce water and energy consumption compared to the pad-steam-dry system.

C.I. Reactive Red 2 (Red 2), commonly used in pad-dyeing, has a dichlorotriazinyl reactive group (Figure S1) which is a very active and hydrolyzable group. Thus, Red 2 was applied for dyeing of cotton fabric with this spraying dyeing system since dichlorotriazinyl reactive group is preferable to form a covalent bonding with cotton cellulose in a short time in comparison to other reactive groups, for example, monochlorotriazinyl reactive group and vinyl sulphone reactive group. Afterwards, the orthogonal design was used to optimize the dye fixation conditions to enhance its inspectability. To our best knowledge, the use of spray pattern dyeing of cotton fabric in the continued dyeing process has not been reported elsewhere. Therefore, we first time report on simultaneous implementation of wet fixation and drying treatment in order to improve dye fixation properties of cotton fabric in the presence of a spray pattern system.

In the present study, the cotton woven fabric was dyed with Red 2 by the spray dyeing equipment, followed by a padding process. After that, a wet heated process was executed, and finally, the drying process was accompanied to fix the dye onto the fabric. The main novelty of this study is to improve the dye fixation rate of cotton fabric with the combination of wet fixation and drying treatment sequentially in a spray pattern process. Besides, the stability of Red 2 in the dye solution is critical and was investigated using the HPLC method. Additionally, the influence of wet fixation and drying conditions on the fixation rate (%) were systematically measured.

## Experimental section

### Materials

Plain weaved scoured and bleached cotton fabric (160 g m^−2^, yarn count 40 s Ne) was supplied by Jiangnan Group Co., Ltd., China. The dyestuff (C. I. Reactive Red 2) was bought in a commercial grade from Shanghai Macklin Biochemical Co., Ltd, China. Nonionic detergent (Luton 500) was purchased from Dalton UK Company. Acetonitrile (HPLC/Spectro) was purchased from Tedia Company (USA). Sodium hydroxide (96%), sodium bicarbonate (> 99.8%), sodium carbonate (99.5%), tetrabutylammonium bromide (99.0%), and ammonium dihydrogen phosphate (99.0%) were purchased from Shanghai Aladdin Biochemical Technology Co., Ltd.

### HPLC analysis of dye solution

To determine dyes stability during the dyeing process, the high-performance liquid chromatography (HPLC) analysis was conducted according to our previous report^28^. Briefly, an L-3000 HPLC C18 column system with 5 µm diameter of the filler particles (250 mm × 4.6 mm, RIGOL, China) was operated at 30 °C throughout the analysis. A gradient method with mobile phase solvents A and B with various proportions (Table S1) was performed using conditions: flow rate of 1.0 mL min^−1^, the detector reference wavelength at 540 nm. The injection sample volume was 20 µL for each analysis.

The Red 2 was dissolved in deionized water (pH 6.8); subsequently, the dye solution pH was maintained between 8.0–13.0 by adding a buffer solution of 0.1 M Na_2_CO_3_ and 0.1 M NaHCO_3_ and a buffer solution of Na_2_CO_3_ (0.1 M) and NaOH (0.1 M), or a NaOH (0.1 M) solution (Table S2) The completely hydrolyzed dye solution was prepared by heating at 100 °C for 2 h at a pH of 13.0. After that, a polytetrafluoroethylene syringe filter (pore size 0.45 µm) was used for solution filtering and an ultrasonic oscillation process adopted for deaeration prior to HPLC measurements.

### Spray dyeing process

**Figure 1:**
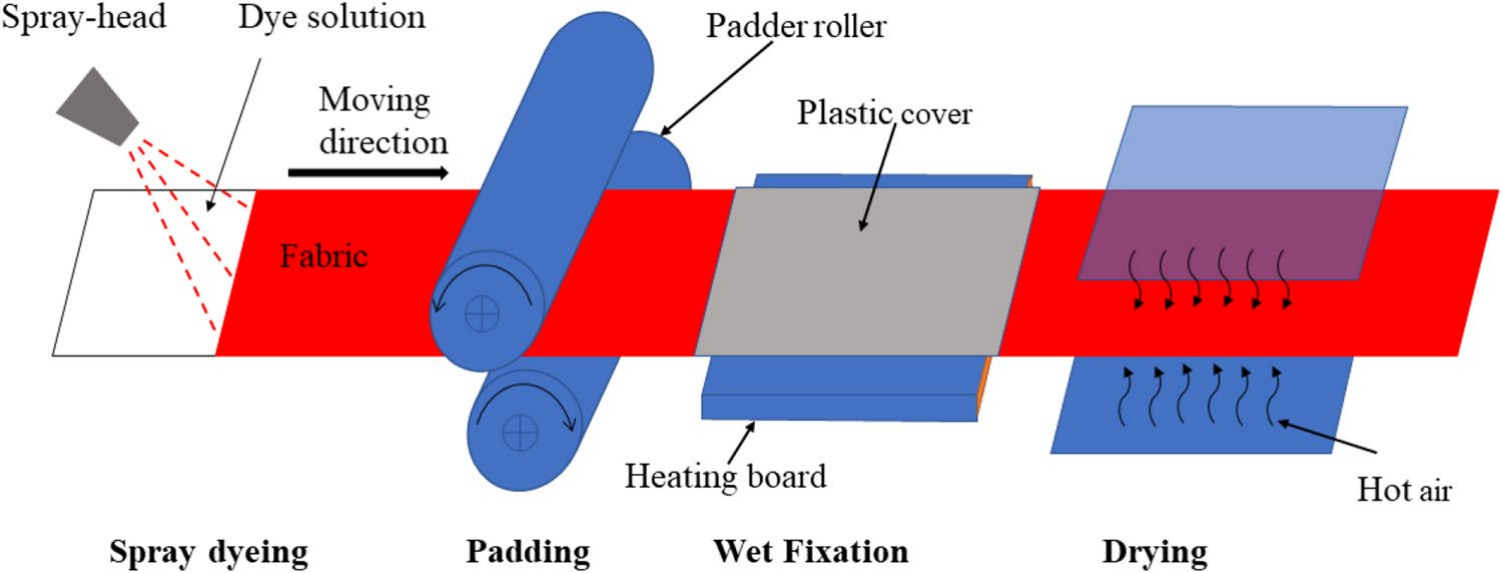
The schematic diagram of the spray dyeing process.

At first, the dye solution of a known concentration was prepared using Red 2. The dye solution pH was adjusted using Na_2_CO_3_ (0.1 M)_,_ NaHCO_3_ (0.1 M)_,_ and/or NaOH (0.1 M), as shown in Table S2. The dyeing process was conducted at room temperature by spray dyeing, followed by a padding process to reach an adsorbed rate of 80% pick-up rate; subsequently, the dyed fabric samples were covered by a plastic film to be wet heated and then dried in an oven (Fig. 1). The dyeing process conditions, including dye concentration (5–30 g L^−1^), pH value of the dye solution (7–12), wet fixation time (0–10 min), and wet fixation temperature (60–100 °C) were varied. After wet fixation, the sample was dried at 70 °C for 6 min. Three specimens of each spray dyed fabric were treated by wet fixation and drying treatments. Moreover, the soaping process was then performed to wash off the unfixed reactive dyes using a rotary infrared laboratory dyeing machine and operated at 95 °C for 15 min at a material-to-liquor ratio of 1:50 with 2 g L^−1^ nonionic detergent. After washing, the dyed samples were subjected to dry for 30 min in an oven chamber at 80 °C. Moreover, the samples color measurements and fastness properties were evaluated and described.

### Orthogonal experimental design

According to the maximum dye fixation rate from the single factor optimizing experiments, each parameters input level was selected for orthogonal experimental design and shown in Table 1. The optimal dyeing process conditions were determined by following “larger the better” S/N ratio analysis (Eq. 1) of the orthogonal array using dye fixation rate (after the combined treatment of wet fixation and drying at 70 °C for 6 min). A professional statistics software, Minitab 20®^29^, was employed to develop the experimental design and analysis.

1 $$S/N=-10 log\left(\frac{1}{n}{\sum }_{i=1}^{n}\frac{1}{{y}_{i}^{2}}\right)$$

where *y*_*i*_ represents the *i*th experimental value and n determines the whole number of experiments.

**Table 1:** L_16_ orthogonal experimental design with their selected parameters and levels.

Parameters	Symbol	Levels			
		Level 1	Level 2	Level 3	Level 4
Wet fixation time (min)	A	3	4	5	6
Wet fixation temperature (°C)	C	50	60	70	80
Dye concentration (g L^−1^)	B	10	15	20	25
Dyeing pH	D	9	10	11	12

### Color strength and color uniformity

The color strength (K/S) values and the L, a and b values (CIE Lab color space values) of detected point were determined using CS-650A spectrophotometer (Hangzhou CHNSpec Technology Co., Ltd., China) and the data collected at 10 nm intervals from 20 random points in the visible wavelength range of 400–700 nm. Afterwards, the average K/S and sum of standard deviation values were calculated, where lower standard deviation values indicate higher color uniformity of the samples^25^. Besides, the colour difference (∆E) of two points in one specimen was calculated using Eq. (2). There are 19 values of delta E for each specimen which is used to investigate the colur uniformity of dyed fabric as well.

2 $$\Delta E=\sqrt{{({L}_{1}-{L}_{n})}^{2}+{({a}_{1}-{a}_{n})}^{2}+{({b}_{1}-{b}_{n})}^{2}}$$

where, L, a, and b are CIE Lab color space values of the detected point; subscript number refers to the detective sequence, and n = 2–20.

### Dye fixation rate

K/S values were used to calculate the fixation rate (F%) of dye using Eq. (3)^30^

3 $$F\%=\frac{(K/S{)}_{a}}{(K/S{)}_{b}}\times 100\%$$

where (K/S)_b_ and (K/S)_a_ are the color strength (K/S) values of the cotton dyed fabric before and after the soaping process, respectively.

### Influence of fixation treatment on the dye hydrolysis

The fabric was dyed by spray using 20 g L^−1^ of Red 2 dye solution at pH 12.0, and the adsorbed dye solution was about 80% pick-up rate. The dyed fabric was treated by wet fixation for 5 min at 60 °C and then dried at 70 °C for 6 min. To detect hydrolysis of Red 2 after the wet fixation treatment and the wet fixation and drying treatment, the treated fabrics were washed in an acidic solution (at pH 4.0) at room temperature, and the residual wash solutions were collected to be analyzed immediately by HPLC and compared to the fresh Red 2 dye solution at pH 12.0. In analyses of the percentage of composite in the dye solutions, only the Red 2 dye with a dichlorotriazinyl group, Red 2 dye with a monochlorotriazinyl group, and Red 2 dye with a completely hydrolyzed group were calculated according to their peak areas in the HPLC chromatogram.

### Colorfastness to washing and rubbing

The colorfastness to washing was evaluated through the use of ISO 105-C06: 2010 test standard in a laboratory fastness tester. The grading of wash fastness was assigned using ISO greyscale by carefully observing the stained dye on the adjacent multi-fiber fabric. The rubbing fastness was investigated by following the ISO 105-X12:2001 standard method, and the grading was determined according to ISO greyscale.

## Results and discussion

### HPLC analysis

**Figure 2:**
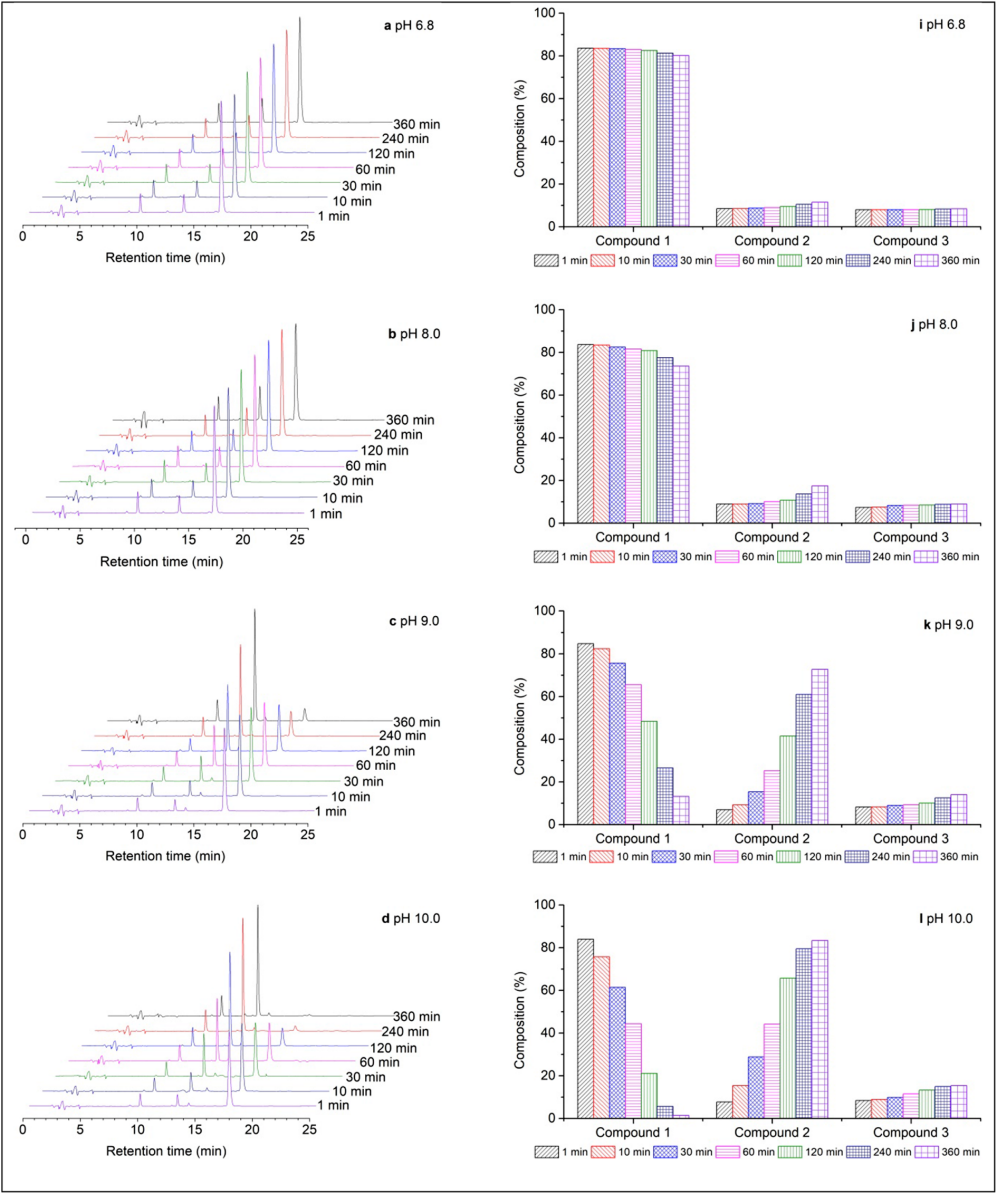

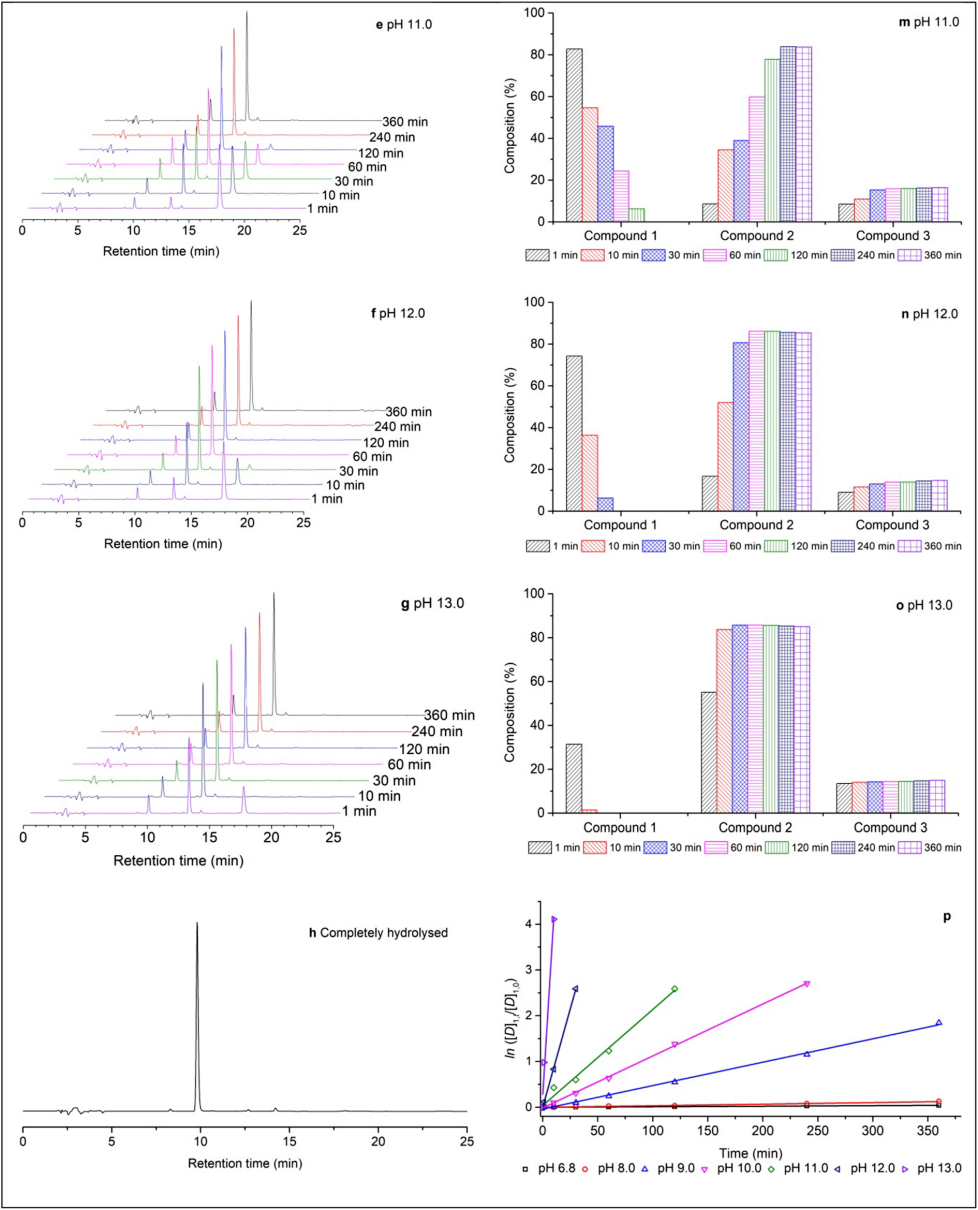
HPLC chromatograms of Red 2 dye solution (**a**–**g**) and the composition percentage of corresponding compounds (**i**–**o**) and the completely hydrolyzed solution (**h**); the pseudo first-order hydrolyzed velocity constant (**p**).

**Figure 3:**
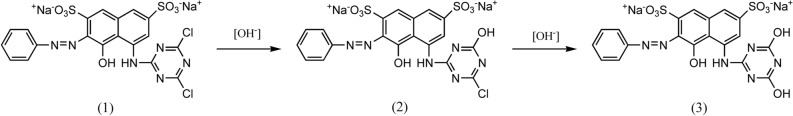
The hydrolysis process of Red 2 in alkaline solution.

The HPLC performances of the diverse dye solutions are shown in Fig. 2. As shown in Fig. 2a, Red 2 dye solution HPLC chromatogram without pH adjustment exhibits three peaks at retention times (t_R_) of 16.573 min, 13.250 min, and 9.613 min. Red 2 has a dichlorotriazinyl group (Compound 1 in Fig. 3) which is very active. During storage, the dichlorotriazinyl group may be hydrolyzed to the monochlorotriazinyl group (Compound 2 in Fig. 3, step 1 of hydrolysis) and the triazine derivative with two hydroxyl groups (Compound 3 in Fig. 3, step 2 of hydrolysis). According to the compounds polarity in Fig. 2, the major peak at t_R_ 16.573 min could be assigned to the Red 2. Moreover, the peaks at t_R_ 13.250 min and t_R_ 9.613 min are attributed to Red 2 dye with one hydroxyl group and Red 2 dye with two hydroxyl groups, respectively. Here, Red 2 dye with two hydroxyl groups refers to the completely hydrolyzed dye^28^. Moreover, the HPLC chromatogram of the completely hydrolyzed dye solution (Fig. 2h) shows that one peak at t_R_ 9.613 min, which corresponds to compound 3 in Fig. 3. In comparison, the other two peaks disappeared.

The composition percentage of the compounds in the Red 2 dye solution under different conditions helps understand the hydrolysis processes. At pH 6.8 (Fig. 2i), the hydrolyzed extent of step 1 was very slight, 0.6% of compound 1 was hydrolyzed to compound 2 after 60 min. With an increase in storage time, the hydrolyzed extent slowly increased by about 3.5% after 360 min. In step 2 of the hydrolysis process, the composition percentage change of compound 3 was negligible. Therefore, it can be concluded that at pH 6.8, the Red 2 in the dye solution was stable in 60 min, and subsequently, slight hydrolysis occurred at step 1 by extending the storage time.

At pH 8.0, the hydrolysis rate was slow, with increased storage time. However, the hydrolyzed extent was acceptable, as only 2.1% of compound 1 was hydrolyzed within 60 min, and subsequently, 10.1% of compound 1 was hydrolyzed within 360 min (Fig. 2j). Compound 3 was produced at pH 8.0, and a 1.6% increment of this compound occurred after 360 min of storage. As the pH value continually increased, it was obvious that the reactive dye hydrolyzed process became drastic^31^. The composition of compound 1 was hydrolyzed quickly, but after 10 min storage, compound 1 was reduced by 1.2%, 7.9%, 29.1%, 47.3%, and 82.3% at pH values of 9.0. 10.0, 11.0, 12.0, and 13.0, respectively (Fig. 2k–o). It can be completely hydrolyzed at pH 11.0 in 240 min, pH 12.0 in 60 min, and pH 13.0 in 30 min. The production of compound 3 at pH 9.0–13.0 increased gradually from 14.0 to 14.9% in 360 min and stayed at this level with negligible increases. These observations could be ascribed to the achievement of the hydrolyzed equilibrium of step 2.

As per the diverse HPLC chromatograms and the transformation of the composition percentages, compared to the reactive properties, the dichlorotriazinyl group is more reactive than the monochlorotriazinyl group implying the monochlorotriazinyl reactive dye requires higher pH conditions for covalent bonding with cellulosic fiber than that of dichlorotriazinyl dye. It means that the dyeing conditions for dichlorotrizainyl dye are unsuitable for monochlorotriazinyl dye. The distinction of the reactive property is also reflected by their hydrolyzed velocities^32^, which were calculated by Eqs. (4 and 5), and the calculated values are shown in Table 2. Besides, there was a substantial volume of water present in the dye solution compared to the dye mass. The addition of a buffer solution adjusted the dye solution pH where the [OH]^−^ could be considered a constant. Therefore, at varying pH conditions, the hydrolyzed constants of compound 1 (Fig. 3) were calculated by Eq. (6)^33^, where the results are depicted in Fig. 2p and Table 2 listed the obtained data.

4 $${v}_{1,t}=d{[D]}_{1,t}/dt$$

5 $${v}_{2,t}=d{[D]}_{2,t}/dt$$

6 $$ln\frac{{[D]}_{1,t}}{{[D]}_{1, 0}}={k}_{1}\times t$$

where v_1,t_ (mmol L^−1^ min^−1^) and v_2,t_ (mmol L^−1^ min^−1^) are the hydrolyzed velocities of compound 1 to compound 2 (step 1) and compound 2 to compound 3 (step 2) at time t (min), respectively. Here, [D]_1,t_ (mmol L^−1^) and [D]_2,t_ (mmol L^−1^) are the concentration decreases of compound 1 and the concentration increase of compound 3 at time t (min), respectively, which were calculated by the ratio of peak area in the HPLC chromatogram; t (min) is the hydrolysis time. Besides, k_1_ (min^−1^) is the pseudo-first-order hydrolyzed velocity constant of Red 2.

**Table 2:** Hydrolyzed velocity and velocity constant of Red 2 dye at varying pH conditions.

pH	6.8	8.0	9.0	10.0	11.0	12.0	13.0
t (min)	360	360	360	240	120	30	10
v_1_ × 10^–3^ (mmol L^−1^ min^−1^)	0.394	1.140	7.952	13.217	26.202	104.810	334.341
v_2_ × 10^–3^ (mmol L^−1^ min^−1^)	0.042	0.179	0.681	1.176	2.696	6.865	24.613
K_1_ (min^−1^)	0.0001	0.0003	0.005	0.0113	0.0214	0.0861	0.4165
R^2^	0.9994	0.9938	0.9973	0.9996	0.9943	0.9997	0.9821

Under the same conditions, the reactive groups higher hydrolyzed velocity indicates that it has a more active property compared to the more lowly hydrolyzed velocity-based reactive groups. In Table 2, v_1_ is always higher than v_2_, especially at strong alkaline conditions, suggesting the dichlorotriazinyl group is more reactive than the monochlorotriazinyl group. Besides, the hydrolyzed velocity of step 1 increased dramatically from 0.394 × 10^–3^ mmol L^−1^ min^−1^ at pH 6.8 in 360 min to 334.341 × 10^–3^ mmol L^−1^ min^−1^ at pH 13.0 in 10 min with increasing pH. For step 2, the hydrolyzed velocity, v_2_, gradually increased, but the increments were lower than that of v_1_ under the same conditions. The hydrolyzed velocity constant of Red 2 increased with increasing pH. The constants increased from 0.0001 min^−1^ at pH 6.8 to 0.4165 min^−1^ at pH 13.0. The results indicate that the hydrolyzed extent of Red 2 significantly increased in strongly alkaline conditions.

### Dye fixation pattern

**Figure 4:**
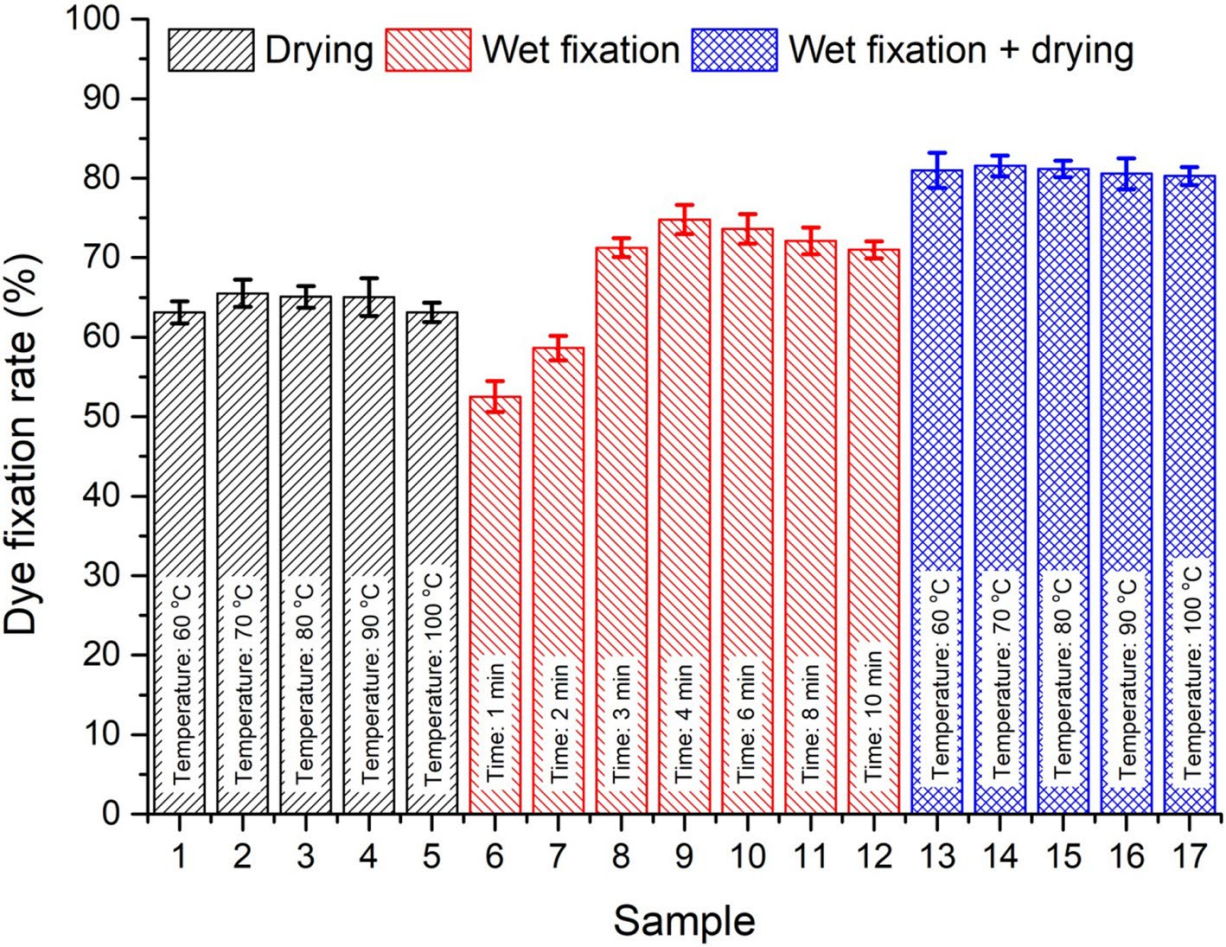
Dye fixation rate of direct drying (sample 1–5), wet fixation treatment for varied times (samples 6–12), and a combination of wet fixation and drying at varied temperatures (samples 13–17).

Based on the designed dyeing technique, after spray dyeing, the dyed cotton fabric progressed through the dye fixation process. Accordingly, there are three methods, including drying, wet fixation, and a combination of wet fixation and drying methods, were evaluated, and the corresponding data are displayed in Fig. 4. The cotton fabric was dyed by spray using a 20 g L^−1^ dye solution at pH 9 with an adsorbed rate of around 80% pick-up rate, and then the dyed samples were directly dried at varying temperatures from 60 to 100 °C for 6 min (samples 1–5), wet fixation treated at 70 °C for a varied time from 1 to 10 min (samples 6–12), or treated by a combination of wet fixation at 70 °C for 4 min and dried at varying temperatures from 60 to 100 °C for 6 min (samples 13–17). For the direct drying pattern, the drying temperature weakly influenced the dye fixation rate, in the range of 60–100 °C, since the dye fixation rates were between 63.11% at 100 °C and 65.52% at 70 °C, with negligible distinction. For the wet fixation treatment, with increased wet fixation time, the fixation rate increased quickly and reached a peak at 74.81% in 4 min; subsequently, it slightly decreased. The combination of wet fixation and drying pattern has the best dye fixation efficiency (above 80%) compared to the direct drying and wet fixation pattern. The drying temperature in the combination pattern showed a negligible distinction on the dye fixation rate, which was similar to the direct drying patterns influence.

The dye fixation rate was influenced by the micro-environment of reactive dye in the fiber structure^9^. At the wet fixation process, the dyes were in wet and warm conditions and formed covalent bonds with cellulosic fiber due to alkali medium presence^34^. During the direct drying treatment, the wet, warm, and alkali conditions damaged quickly, and the beneficial conditions of the reaction existed only for a short time^35^. Thus, the dye fixation rates of direct drying at 60 to 100 °C were similar. Furthermore, the dyed cotton fabric treated by a combination of wet fixation and drying exhibited the best fixation efficiency. This was possible as the covalent reaction occurred first in the wet fixation treatment, and the reaction was further enhanced during the drying process^36^. Meanwhile, as the drying process began, the water volume in the dyed fiber was slowly evaporated, *i.e.,* the liquid volume in the dyeing micro-environment decreased, causing dye promotion of the dye-liquid in the fiber pores to contact the fiber, as well as producing stronger alkali conditions. These changes in conditions contributed to an improved dye fixation rate^16^.

**Figure 5:**
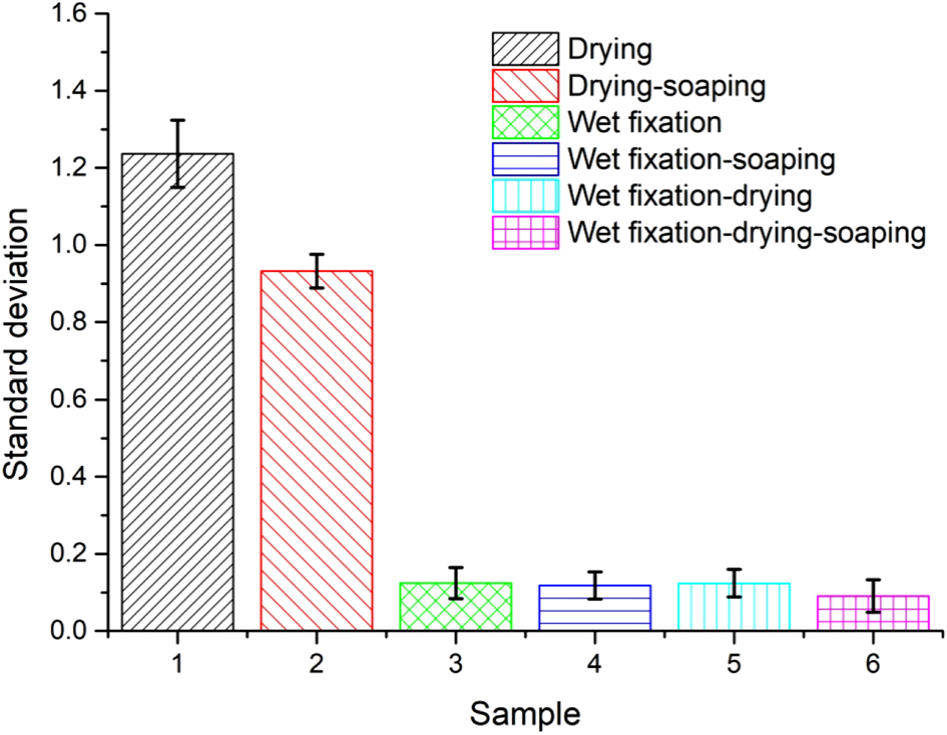
Color uniformity (standard deviation of K/S) of samples treated by fixation treatments.

**Figure 6:**
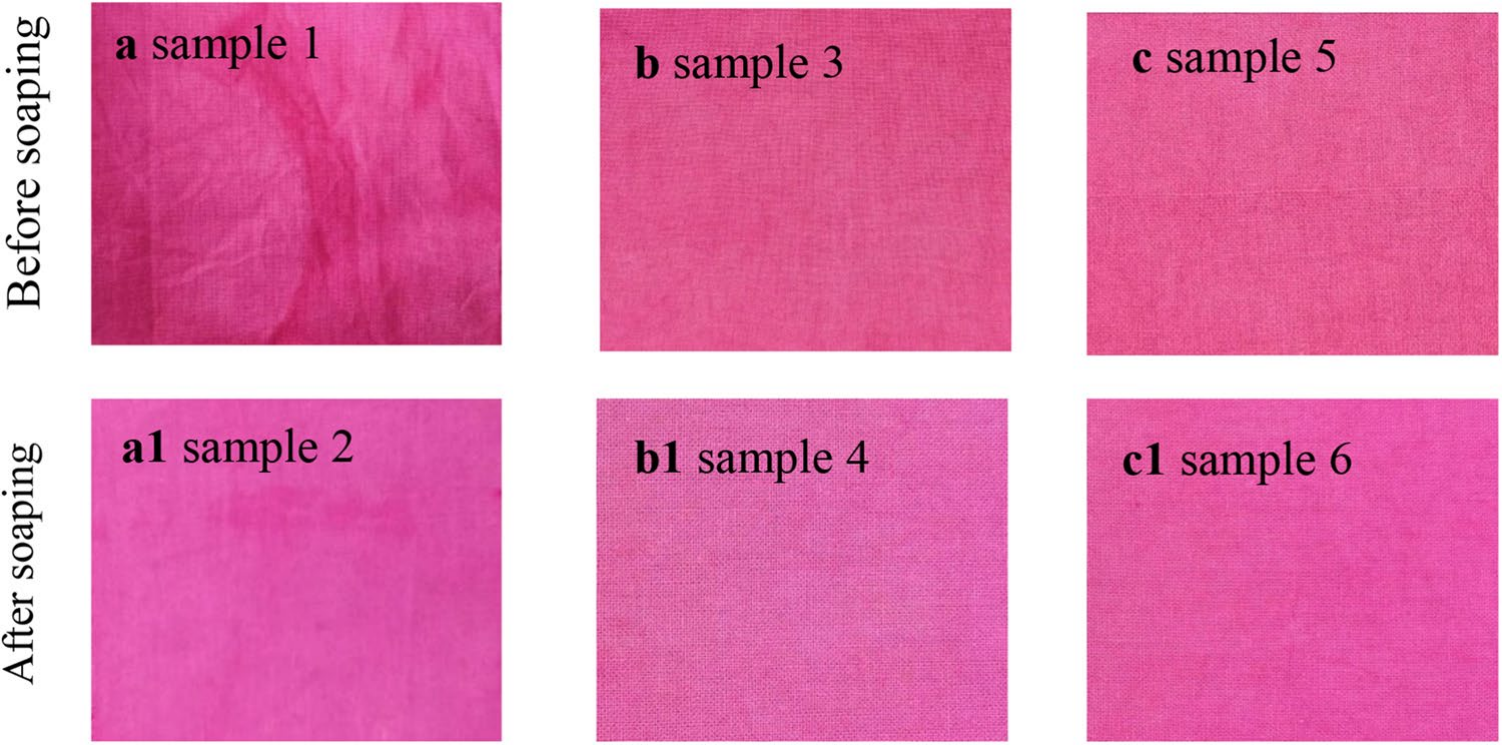
The color shade of dyed samples treated by (**a**) drying, (**b**) wet fixation, and (**c**) the combination of wet fixation and drying before and after soaping.

Lastly, during the direct drying process, the fibers liquid content was evaporated, accompanying dye migration from dry to wet fields, resulting in uneven color. The color uniformities of the dyed samples are shown in Figs. 5 and 6. Samples 1 and 2 were directly dried at 70 °C for 6 min, samples 3 and 4 (Fig. 5) were treated by wet fixation at 70 °C for 4 min, and samples 5 and 6 were treated by wet fixation at 70 °C for 4 min and subsequently dried at 70 °C for 6 min. It showed that the direct drying pattern to the pad-dyed samples causes a higher value of the standard deviation of K/S values, while for both the wet fixation and wet fixation-drying patterns, the standard deviation remarkably decreased, especially for sample 6 (wet fixation-drying-soaping), where its standard deviation value was as low as 0.09, compared to that of 0.93 for sample 2 (drying-soaping). Meanwhile, the soaping process could improve the samples colour uniformity since the unfixed dye in the dyed fabric was possibly dissolved in the soaping solution and re-stained in the fabric^30^.

**Figure 7:**
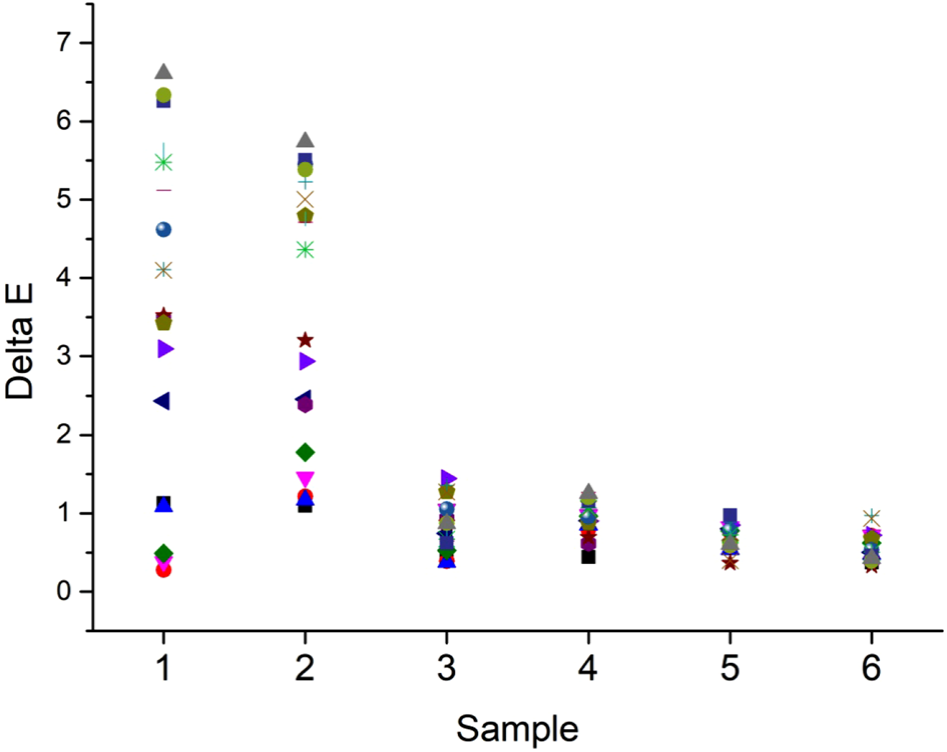
Color uniformity (delta E values) of dyed sampels treated by (**a**) drying, (**a1**) drying-soaping, (**b**) wet fixation, (**b1**) fixation-soaping, (**c**) wet fixation-drying, and (**c1**) wet fixation-drying-soaping.

In evaluation of colur uniformity, the color tolerance is considered as well, and it is assessed by the delta E value (Eq. 1). Normally, the color tolerance is accepted when the delta E is smaller than 1; sometimes, the delta E requires a lower than 0.8. The colour parameters (L, a, and b values) of detected spots in each dyed specimen were recorded and used to analyse the color difference (delta E), which is shown in Fig. 7. Samples 1 and 2 showed bigger ranges of detal E, and the standard deviation values of 19 delta E values were 2.11 and 1.72 for samples 1 and 2, respectively. In comparison, the delta E values ranges of samples 3 and 4 dramatically shortened and their standard deviation values were 0.33 and 0.22, respectively. A further shortened range of delta E was displayed on samples 5 and 6, and the corresponding standard deviation values were 0.17 and 0.19, respectively. The short-range of delta E implied that the dyed sample had a more uniform color shade. Thus it exhibited that the soaping process contributed to color uniformity. However, only samples 5 and 6 had all delta E values that were smaller than 1, which indicated that the color uniformity of both samples was acceptable. Thus, the dyed fabric first treated by the wet fixation process and then dried was effective in avoiding the dye migration problem of direct drying fixation, as most of the reactive dyes were fixed in the wet fixation process.

In summary, the combination fixation pattern, which is the wet fixation at 70 °C for 4 min and drying at 70 °C for 6 min was applied to fix the dyestuff into dyed fabric after the spray dyeing.

### Influence of wet fixation time on dye fixation rate

**Figure 8:**
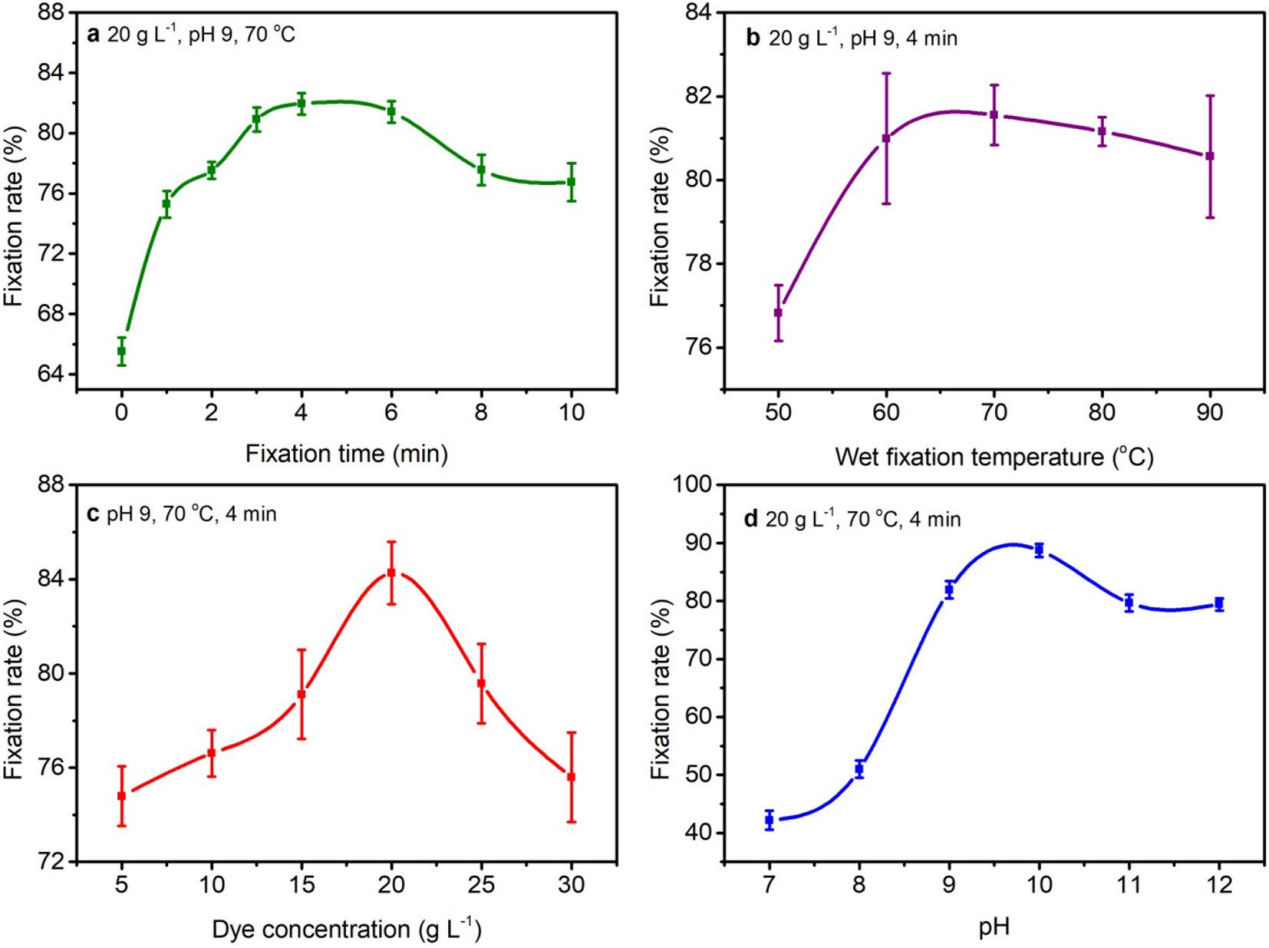
Influence of (**a**) wet fixation time, (**b**) wet fixation temperature (**c**) dye concentration, and (**d**) dye solution pH on fixation rate (%) of reactive dye on cotton fiber.

The fabrics were dyed with 20 g L^−1^ dye at pH 9, and then the dyed fabric sample was wet treated at 70 °C over a varied wet fixation time from 0 to 10 min. Consequently, the dyed wet treated fabric was dried at 70 °C for 6 min. The influence of wet fixation time on the dye fixation rate is exhibited in Fig. 8a. It was noticed that the dye fixation rate increased from 0 to 4 min, and the maximum fixation rate was 81.95% at 4 min. However, when the wet fixation time was more than 4 min, the bonding between fiber and dye may break, the dye hydrolysis increased, and the fixation rate gradually decreased^37^. Therefore, four minutes was found as the optimal wet fixation time to fix reactive dye onto cotton fiber.

### Influence of wet fixation temperature on dye fixation rate

The effect of wet fixation temperatures on dye fixation rate was studied, and the suitable fixation temperature was determined. Therefore, the fixation rate and dye hydrolysis were optimized under economic conditions. The fabrics were dyed with 20 g L^−1^ dye at pH 9, then the fabric was wet treated for 4 min. The wet fixation temperatures varied from 50–90 °C. Consequently, the fabric was subjected to dry at 70 °C for 6 min. It can be seen from Fig. 8b, the dye fixation rate enhanced with increasing temperature until 70 °C. However, at higher temperatures (> 70 °C), the fixation rate started to decrease. Among the wet fixation temperatures (50–90 °C), the highest dye fixation rate was noticed at 70 °C. This was because 70 °C may contribute to fiber swelling and dye penetration into the fiber structure^37^. Therefore, the reaction rate between the fiber and reactive dyes increased at 70 °C, while the dyes hydrolyze above 70 °C^37^. Hence, according to the analysis, 70 °C was the favored wet fixation temperature for reactive dyes.

### Influence of dye concentration on dye fixation rate (%)

The fabric was dyed in the presence of various dye concentrations in the range of 5–30 g L^−1^ at pH 9, then wet treated at 70 °C for 4 min, and dried at 70 °C for 6 min. Figure 8c shows that the fixation rate increases with a higher dye concentration range from 5 to 20 g L^−1^, and afterwards, the fixation rate started decreasing with increased dye concentration. The maximum fixation rate was observed at 20 g L^−1^ of dye concentration. At a dye concentration of 20 g L^−1^, maximum dye molecules can easily penetrate into the fiber, resulting in dye-fiber bonding. Moreover, when the dye concentration exceeds 20 g L^−1^, the dye aggregation was higher, and most of the dye molecules did not penetrate into the fiber, so they could not generate enough space to bond with the fiber^38^. Therefore, 20 g L^−1^ of dye concentration was advised for the maximum reactive dye fixation rate in spray-wet fixation-dry dyeing of cotton fabric.

### Influence of dyeing pH on dye fixation rate (%)

In reactive dyeing, pH is a key factor in achieving higher dye fixation rates. In Fig. 8d, the fixation rate (%) result is shown concerning the influence of pH on the dye solution. The fabric was dyed with 20 g L^−1^ dye at pH 7–12, then dyed fabric was wet treated at 70 °C for 4 min. Afterwards, the fabric was dried at 70 °C for 6 min. Alkaline conditions were favorable for bonding between reactive dyes and cellulosic fibers, and the fixation rate increased with increasing pH values from 7 to 10. The maximum dye fixation rate was observed at pH 10 (Fig. 8d), which may be attributed to low dye hydrolysis^39^. However, when the pH value exceeded 10, the fixation rate decreased because higher pH is feasible for dye hydrolysis,, while more alkaline conditions may retard the reaction between the dye and fiber^39^.

### Orthogonal array and optimal processing conditions

Optimal dyeing conditions were selected based on the S/N ratio (Eq. 1) analysis of the orthogonal array. As a higher fixation rate is identified as a suitable dyeing performance, the better function was nominated during the S/N ratio analysis. The maximum value of each factor S/N ratio suggests the individual factors optimal level. The experimental results of K/S value, fixation rate, and fixation rate S/N ratio values are shown in Table 3.

**Table 3:** Details results of L_16_ orthogonal design.

Exp. no	A (min)	B (°C)	C (g L^−1^)	D	(K/S)_b_	(K/S)_a_	Fixation rate (%)	S/N ratio (dB)
1	3	50	10	9	3.98	2.79	70.10	36.91
2	3	60	15	10	5.42	4.59	84.67	38.55
3	3	70	20	11	6.65	5.54	83.31	38.41
4	3	80	25	12	7.69	6.52	84.78	38.57
5	4	50	15	11	5.47	4.66	85.19	38.61
6	4	60	10	12	3.94	3.33	84.52	38.54
7	4	70	25	9	7.38	6.09	82.52	38.33
8	4	80	20	10	5.85	4.86	83.08	38.39
9	5	50	20	12	6.21	5.81	93.56	39.42
10	5	60	25	11	7.11	6.5	91.42	39.22
11	5	70	10	10	4.02	3.36	80.09	38.07
12	5	80	15	9	5.19	4.02	80.92	38.16
13	6	50	25	10	7.56	6.03	79.76	38.04
14	6	60	20	9	6.42	5.05	78.66	37.92
15	6	70	15	12	5.46	4.65	85.16	38.60
16	6	80	10	11	4.44	3.53	79.95	38.06

The response table of parameters and levels with delta statistics were calculated using the experimental result, as listed in Table 4. The delta characteristics can be defined by measuring the difference between the highest and lowest average values of each factors S/N ratio. The obtained values were found to be in ascending order, starting with rank 1 (highest value), rank 2 (second highest value) and so on.

**Table 4:** Response table data with S/N ratio of dye fixation rate.

Level	Wet fixation time (min)	Wet fixation temperature (°C)	Dye concentration (g L^−1^)	Dyeing pH
1	38.11	38.24	37.90	37.83
2	38.47	38.56	38.48	38.26
3	38.72	38.36	38.54	38.57
4	38.15	38.29	38.54	38.78
Delta	0.61	0.31	0.64	0.95
Rank	3	4	2	1
Optimum level	A_3_	B_2_	C_3_	D_4_

**Figure 9:**
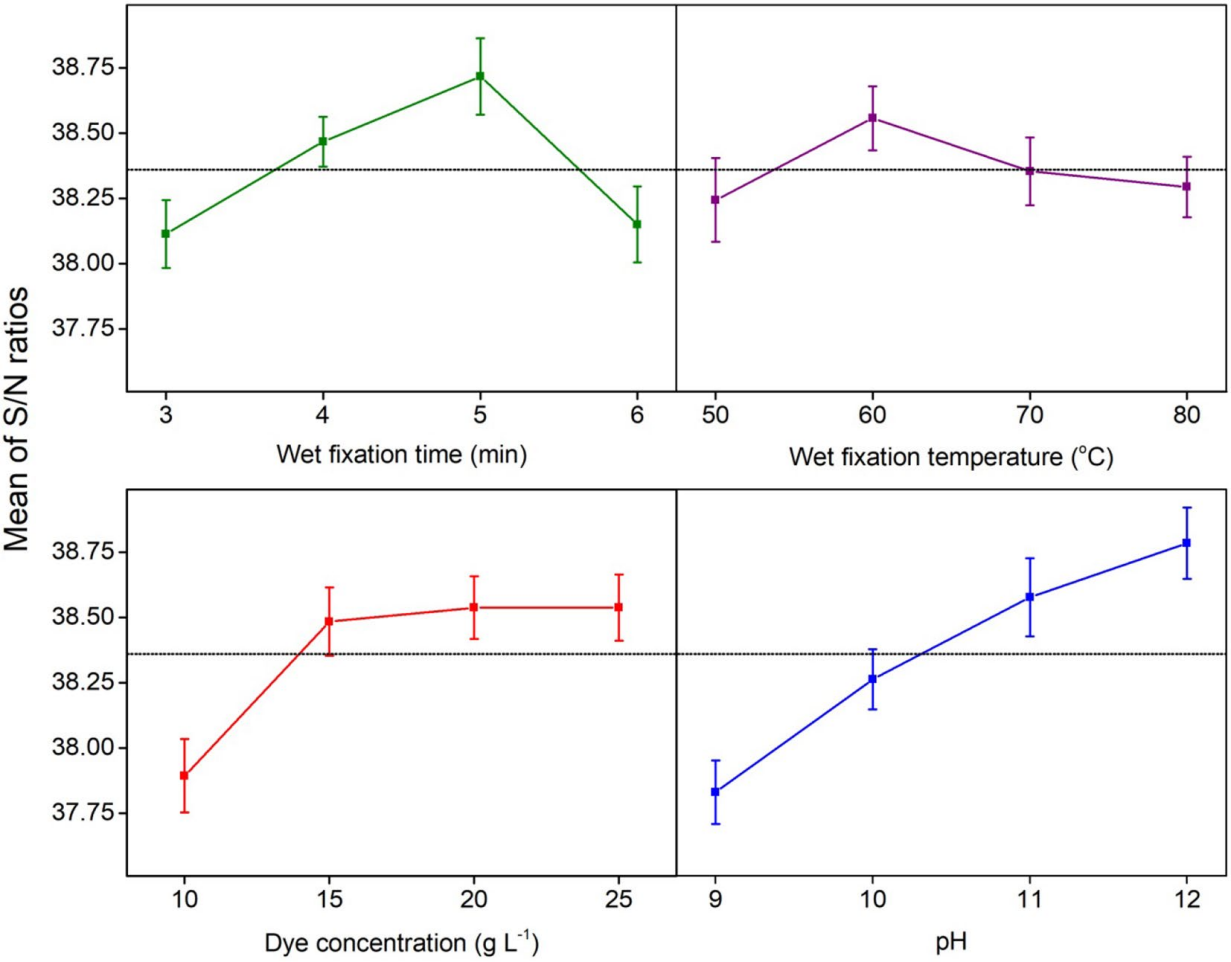
The main effects plot for each factor with their mean S/N ratios.

The dyeing pH had the maximum effect on the dye fixation rate having a maximum delta value of 0.95 and pH ranked 1. Next were dye concentration, wet fixation time, and wet fixation temperature with delta values of 0.64 (rank 2), 0.61 (rank 2), and 0.31 (rank 3), respectively. In Fig. 9, the main effects plot of the S/N ratio for different factors is presented. The fourth level value of dyeing pH, the second level value of wet fixation temperature, and the value of the third level of wet fixation time and dye concentration reached the maximum dye fixation rate. The S/N ratio values of 38.72 dB (A_3_), 38.56 dB (B_2_), 38.54 dB (C_3_), and 38.78 dB (D_4_) for wet fixation time, wet fixation temperature, dye concentration, and dyeing pH, respectively also provided the same optimum conditions for the maximum fixation rate (Table 4).

It was noticed that when the wet fixation time increased from 3 to 5 min, the dye fixation rate increased. As the fixation time further increased (6 min), the fixation rate decreased as the bonding between fiber and dye may break and the dye hydrolysis increases^37^. Therefore, five min wet fixation time was found as the optimal time for the dye fixation reaction. The result of this study for wet fixation temperature shows that the dye fixation rate increases with increasing temperature in the range of 50 to 60 °C because of the fiber swelling and penetration of dyes into the fiber structure. Hence, increasing the wet fixation temperature above 60 °C can reduce the fixation reaction and fixation rate, and increase dye hydrolysis^37^. Therefore, 60 °C temperature was found to be the optimal temperature for the maximum fixation rate.

With increasing dye concentration (10–20 g L^−1^), the fixation rate increased. In particular, at dye concentration enhanced from 20 to 25 g L^−1^, the fixation rate showed a similar trend and it should be pointed out that at higher concentration (25 g L^−1^), the dye molecules could not get enough space to bond with the fiber^38^. Therefore, 20 g L^−1^ was found to be the optimal dye concentration from the orthogonal analysis. Under alkaline conditions, with increasing the dye solution pH, the reaction between the reactive dye and cellulosic fiber was enhanced, and the fixation rate also increased. Therefore, the maximum dye fixation rate was found at pH 12. Consequently, from the orthogonal analysis, optimal conditions were determined under conditions of 5 min of wet fixation time, 60 °C of wet fixation temperature, 20 g L^−1^ of dye concentration, and pH 12 of dye solution.

In the orthogonal experiments, the dye solution was freshly prepared and used immediately, thus the dye fixation performance was not influenced by the hydrolyzed property, i.e., the hydrolysis of Red 2 in dyebath before padding is negligible. According to the stability of Red 2 at different pH conditions, Red 2 was hydrolyzed quickly at pH 12 (Fig. 2n). Therefore, it is not recommended to prepare the Red 2 stock solution at pH 12. To investigate the optimal dyeing conditions for the spray machine, a dye stock solution was prepared at pH 6.8, and then the dye stock solution pH value was increased to 12 by dosing with alkali. After that, the prepared solution (20 g L^−1^) immediately spray-dyed the fabric with 80% pick-up rate of adsorption rate, and then treated with wet fixation at 60 °C for 5 min, followed by drying at 70 °C for 6 min.

The final samples dyeing performances, such as K/S values (before and after the soaping process), dye fixation rate, color uniformity (after soaping), and colorfastness are shown in Table 5. The fabric was dyed with 20 g L^−1^ dye at pH 12, followed by a wet fixation treatment at 50 °C (sample 1) and 60 °C (sample 2) for 5 min, and then dried at 70 °C for 6 min. The dye fixation rates of samples 1 and 2 are 93.56% and 94.42%, respectively. This proves that the optimal conditions obtained by the orthogonal analysis using Minitab software are precise. The samples showed good colorfastnesses to washing (grade between 4–5) and dry rubbing (grade between 4–5). These phenomena can be ascribed to the good dye fixation and the soaping process contribution since the soaping process gave a high efficiency of removing the unfixed dyestuff from the dyed fabric surface, resulting in overall colorfastness performance. However, the wet rubbing fastness (grade 3–4) was found at a considerable level.

**Table 5:** Characterization of the dyed fabrics.

Sample	(K/S)_b_	(K/S)_a_	Fixation rate (%)	σ(λ)	Color fastnesses (Grade)		
					Washing	Dry rubbing	Wet rubbing
1	6.21	5.81	93.56	0.17	4–5	4–5	3–4
2	6.81	6.43	94.42	0.17	4–5	4–5	3–4

### Influence of wet fixation and drying treatments on dye hydrolysis

**Figure 10:**
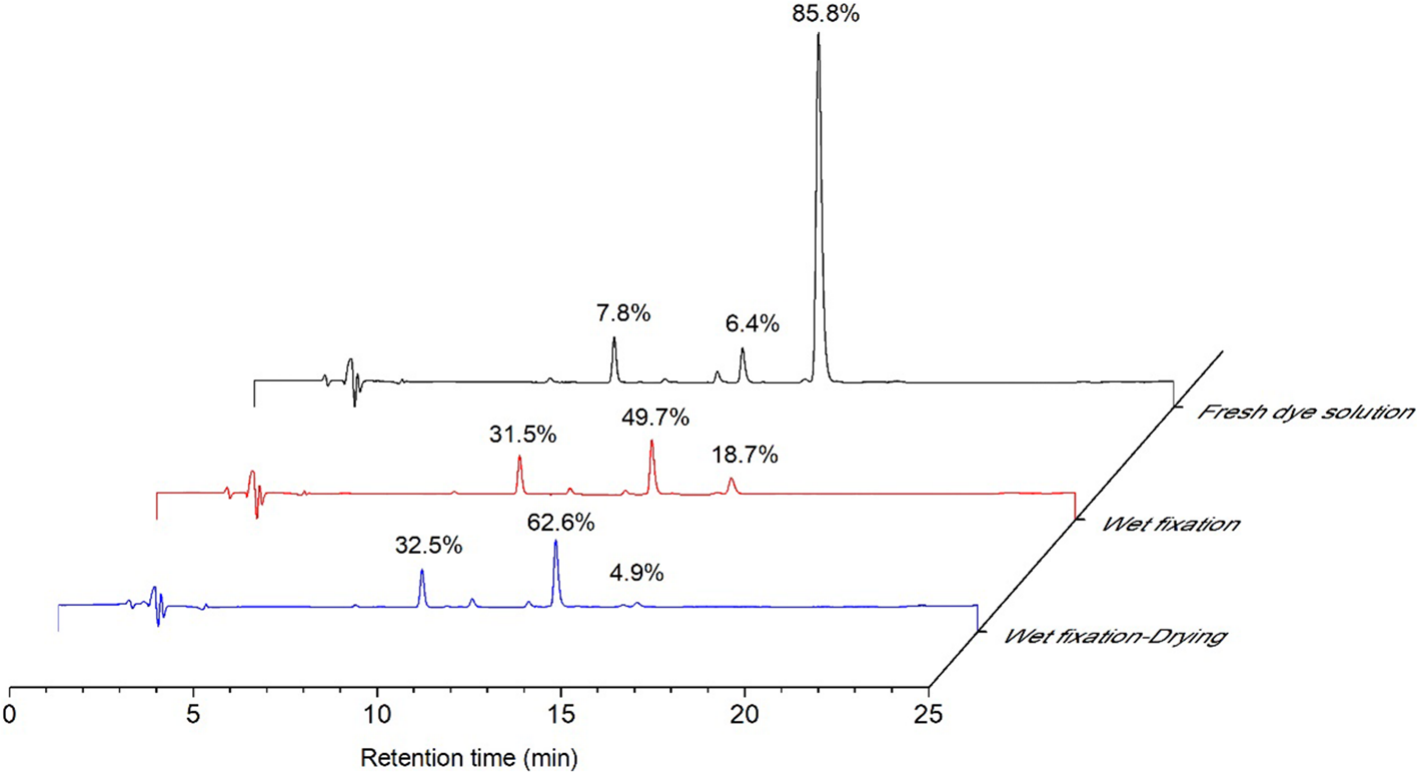
HPLC chromatograms of the Red 2 dye after wet fixation and wet fixation-drying treatment.

To inspect the influence of wet fixation treatment and drying process on the dye hydrolysis, the dyed fabrics, which were treated by either wet fixation process or wet fixation-drying process, were washed in distilled water, and the corresponding washed dye solutions were then measured by HPLC, and the corresponding results are listed in Fig. 10. As shown, 85.8% of the Red 2 dye was found in the fresh dye solution containing dichlorotriazine group, 6.4% in the Red 2 dye solution containing monochlorotriazine group, and 7.8% in the Red 2 dye solution containing complete hydrolyzed group. After wet fixation treatment of the dyed fabric, the Red 2 dye in the washed dye solution containing the dichlorotriazine group decreased to 18.7%, while the Red 2 dye containing the monochlorotriazine group increased to 49.7%, and the dye containing a completely hydrolyzed group increased to 31.5% because the Red 2 dye not only reacted with cellulosic fiber but also hydrolyzed. After the combination of wet fixation and drying treatment, the Red 2 dye in th washed dye solution with dichlorotriazine group reduced to 4.9%, while the Red 2 dye with monochlorotriazine group increased to 62.6%, and the dye containing a completely hydrolyzed group increased to 32.5%. The results reveal that after wet fixation and drying treatment, the Red 2 dye containing the dichlorotriazine group almost disappeared, and the main composite was the Red 2 dye containing the monochlorotriazine group.

## Conclusion

The dyeing of cotton fabric using a spray dyeing machine, as well as dye fixation treatment, were studied. The dye stability in the dye stock solution reduced with increased pH values of the dye solution. The dye was almost completely hydrolyzed within 10 min at pH 13. The combination of wet fixation and drying process showed maximum dye fixation rate and excellent color uniformity properties compared to samples with direct drying and only wet fixation treatment. The optimal process conditions of spray dyeing and a combination of wet fixation and drying were determined using an orthogonal array (wet fixation: wet fixation time 5 min, wet fixation temperature 60 °C, dye concentration 20 g L^−1^, dye solution pH 12; drying: at 70 °C for 6 min), and under the optimum conditions, the fixation rate was 94.42%. After a combination of wet fixation and drying treatments, the Red 2 dye with monochlorotriazine group was the main compound instead of the dichlorotriazine group, indicating that fixation treatments had an effect on the dye structure with hydrolysis phenomena. The colorfastness properties of the dyed samples (wash fastness: Grade 4–5; dry/wet rubbing fastness: Grade 4–5/Grade 3–4) were observed. Thus, these results will encourage carrying out additional experiments with a new system in the presence of other kinds of dyes such as direct dye, acid dye, base dye, and so on to improve dye fixation rate onto cotton fabric using this spray dyeing technology. Moreover, the developed dyeing process may reduce chemicals and water consumption in textile industries.

## Supplementary Information

Supplementary Information.

## Data Availability

The datasets generated during the current study are available from the corresponding author on reasonable request (Prof. Yingjie Cai, Y. Cai).

## Code availability

No code was created during the current manuscript.
