# Supplementary material for: Combination of wet fixation and drying treatments to improve dye fixation onto spray-dyed cotton fabric
Source: Sci Rep. 2021 Jul 28;11:15403. doi: 10.1038/s41598-021-94885-z (PMC8319435; doi:10.1038/s41598-021-94885-z)
Supplement: Supplementary file 1 — Supplementary Information. [file 41598_2021_94885_MOESM1_ESM.docx]

**Supporting Information**

**Combination of wet fixation and drying treatments to improve dye fixation onto spray-dyed cotton fabric**

Lina Lin^‡1,2^, Wenju Zhu^‡1,2^, Cong Zhang^1,2^, Md. Yousuf Hossain^1,2^, Zubair Bin Sayed Oli^1,2^,

Md. Nahid Pervez^1,3^, Shamima Sarker^4^, Md. Ikram Ul Hoque^5,6^, Yingjie Cai^1,2 *^, Vincenzo Naddeo^3^*

*^1^ Hubei Provincial Engineering Laboratory for Clean Production and High Value Utilization of Bio-based Textile Materials, Wuhan Textile University, Wuhan, 430200, China*

*^2^ Engineering Research Centre for Clean Production of Textile Dyeing and Printing, Ministry of Education, Wuhan Textile University, Wuhan, 430200, China*

*^3^ Sanitary Environmental Engineering Division (SEED), Department of Civil Engineering, University of Salerno, via Giovanni Paolo II 132, 84084 Fisciano (SA), Italy*

*^4^ College of Material Science and Engineering, Donghua University, Shanghai, 201620, China*

*^5^ Discipline of Chemistry, The University of Newcastle, University Drive, Callaghan, NSW 2308, Australia.*

*^6^ Australian Institute for Bioengineering and Nanotechnology (AIBN), The University of Queensland, Brisbane, QLD 4072, Australia*.

*^‡^These authors contributed equally to the work*

** Corresponding Authors:*

*Prof. Yingjie Cai (yingjiecai@wtu.edu.cn) ; Prof. Vincenzo Naddeo (vnaddeo@unisa.it)*





**Figure S1.** Chemical structure of C.I. Reactive Red 2

**Table S1.** HPLC analysis of Red 2 by following the gradient elution process

| Time (min) | Solvent A^*^ (%) | Solvent B^*^ (%) |
| --- | --- | --- |
| 0 | 55 | 45 |
| 10 | 45 | 55 |
| 10 | 45 | 55 |
| 25 | 55 | 45 |

*Solvent A: 0.025 mmol L^-1^ of tetrabutylammonium bromide and 0.01 mol L^-1^ of ammonium dihydrogen phosphate. Solvent B: acetonitrile.

**Table S2.** pH value of the dye solution

| Na_2_CO_3_ (mL) | NaHCO_3_ (mL) | NaOH (mL) | pH |
| --- | --- | --- | --- |
| 5 | 95 | / | 9.0 |
| 60 | 40 | / | 10.0 |
| 90 | 10 | / | 11.0 |
| 90 | / | 10 | 12.0 |
| / | / | 100 | 13.0 |
